# Drivers of reef shark abundance and biomass in the Solomon Islands

**DOI:** 10.1371/journal.pone.0200960

**Published:** 2018-07-30

**Authors:** Jordan S. Goetze, Tim J. Langlois, Joe McCarter, Colin A. Simpfendorfer, Alec Hughes, Jacob Tingo Leve, Stacy D. Jupiter

**Affiliations:** 1 Department of Environment and Agriculture, Curtin University, Bentley Campus, Western Australia, Australia; 2 Marine Program, Wildlife Conservation Society, Bronx, New York, United States of America; 3 The UWA Oceans Institute and School of Biological Sciences (M470), Faculty of Science, The University of Western Australia, Crawley, Western Australia, Australia; 4 Center for Biodiversity and Conservation, American Museum of Natural History, New York, United States of America; 5 Wildlife Conservation Society, Melanesia Regional Program, Suva, Fiji; 6 Centre for Sustainable Tropical Fisheries & Aquaculture, and College of Marine and Environmental Sciences, James Cook University, Townsville, Queensland, Australia; 7 Coastal and Marine Management, Munda, Solomon Islands; Department of Agriculture and Water Resources, AUSTRALIA

## Abstract

Remote island nations face a number of challenges in addressing concerns about shark population status, including access to rigorously collected data and resources to manage fisheries. At present, very little data are available on shark populations in the Solomon Islands and scientific surveys to document shark and ray diversity and distribution have not been completed. We aimed to provide a baseline of the relative abundance and diversity of reef sharks and rays and assess the major drivers of reef shark abundance/biomass in the Western Province of the Solomon Islands using stereo baited remote underwater video. On average reef sharks were more abundant than in surrounding countries such as Fiji and Indonesia, yet below that of remote islands without historical fishing pressure, suggesting populations are relatively healthy but not pristine. We also assessed the influence of location, habitat type/complexity, depth and prey biomass on reef shark abundance and biomass. Location was the most important factor driving reef shark abundance and biomass with two times the abundance and a 43% greater biomass of reef sharks in the more remote locations, suggesting fishing may be impacting sharks in some areas. Our results give a much needed baseline and suggest that reef shark populations are still relatively unexploited, providing an opportunity for improved management of sharks and rays in the Solomon Islands.

## Introduction

Worldwide, many shark and ray populations are declining as a result of overexploitation by fisheries [1–3]. Most shark species are slow growing, take several years to reach sexual maturity, and produce few young, making them highly vulnerable to overfishing [4]. Globally, total shark mortality needs to be reduced in order to rebuild depleted populations [5], but conservation needs vary dramatically between nations [6,7]. Remote island nations face a number of challenges in addressing concerns about shark population status, including access to rigorously collected data and resources to manage fisheries. Despite this, there is a small but growing body of knowledge on the status of sharks in these areas [8,9].

Fishing pressure in the Solomon Islands is largely driven by population pressure and market access, which have significant impacts on targeted fish and invertebrate communities [10,11]. Subsistence fishers in the Solomon Islands traditionally target shallow-water estuarine and reef fish, with only the occasional shark kept for local consumption or sale [12]. However, sharks are directly targeted by some industrial longline vessels and caught as by-catch when targeting tuna [13]. These commercial fisheries primarily target sharks for their fins as they can be dried and stored, creating a profitable fishery despite sporadic markets and infrequent opportunities for international export [13].

The indigenous people of the Solomon Islands, like other Melanesian countries, have complex systems of customary marine tenure (CMT) under which fishing rules can be flexibly applied and adjusted to regulate access to marine resources [14], though the nature of CMT systems and those involved in decision-making vary across the country [15]. Since the 1990s, community-based resource management (CBRM) and community-based co-management approaches have been promoted to build on CMT arrangements to manage small-scale coral-reef fisheries, including reef sharks [10,16]. While CBRM measures around the western Pacific have demonstrated some success in maintaining fish populations [17], protection of highly vulnerable and mobile shark populations is likely to require additional top-down controls, such as no-take areas or catch bans [18]. Some top down controls are being considered by the Solomon Islands Government, who have developed a draft National Plan of Action for sharks. For example, the draft plan makes recommendations for: catch restrictions where monitoring data indicate populations are threatened; and consideration of moratoria during breeding seasons or no-take areas over known breeding habitats (S. Jupiter, pers. comm.).

Baited remote underwater video systems (BRUVs) offer a non-destructive alternative to fishing surveys (e.g. longlines), while providing similar estimates of shark abundance [19]. The maximum number of each species seen at any one time during a video (MaxN; [20]) provides a conservative estimate of abundance, though limits BRUV surveys to relative estimates rather than densities. The use of bait increases the proportion of predatory species observed [21–23] and overcomes the issue of small sample sizes, often associated with the use of diver-based surveys when sampling sharks [24]. BRUVs have been used to survey a wide variety of sharks, across a broad range of locations, to answer a diverse range of ecological questions [25–30]. However, to provide a comprehensive assessment of the entire fish assemblage, a combination of both BRUVs and diver-based surveys is recommended, particularly for questions related to herbivorous and invertivorous fishes [22,23,31].

At present, very little data are available on shark populations in the Solomon Islands, and specific scientific surveys to document shark and ray diversity and distribution have not been completed [32]. In support of national efforts to manage sharks, we assess the relative abundance and biomass of reef sharks across four locations in the Solomon Islands using stereo baited remote underwater video systems (stereo-BRUVs). Stereo-BRUV surveys of shark and rays have been completed across the Indo-Pacific (e.g. Fiji [26], Indonesia [30] and New Caledonia [33]), which will allow our results to be placed into context. We aimed to provide a baseline of the relative abundance and diversity of reef sharks and rays and assess the major drivers of reef shark abundance/biomass in the Solomon Islands, including the influence of habitat type/complexity, depth, location and prey biomass (collected using diver-based surveys).

## Material and methods

### Study area

**Fig 1:**
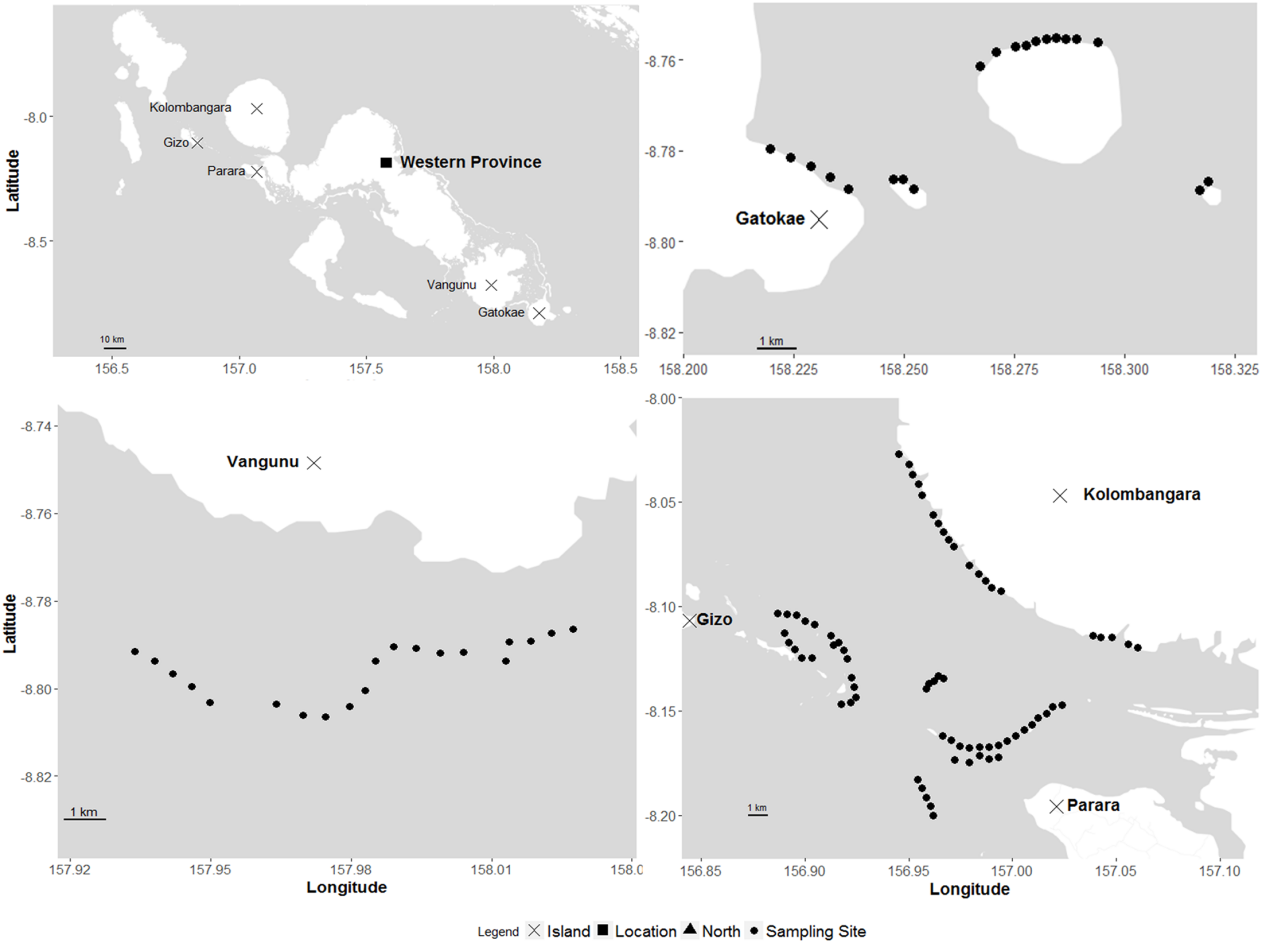
Map of survey locations across the Western Province of the Solomon Islands.

Sampling occurred between October 17—November 1, 2015, in four main locations: western Kolombangara Island (*Kolombangara)*, north-western Parara and Gizo islands (*Parara/Gizo*), southern Vangunu Island (*Vangunu*), and eastern Gatokae and adjacent smaller islands (*Gatokae*) in the Western Province, Solomon Islands (Fig 1). The sites encompass a range of geographic and ecological features. Kolombangara, Gatokae and Vangunu are high, steep volcanic islands with major river systems and associated sediment outflow. The southwestern reefs adjacent to Kolombangara have a narrow fringe and steep drop-offs. Most of the sites sampled around Parara and Gizo islands are sheltered from large wave exposure, with the exception of the five sites on the western barrier reef off Parara Island (Fig 1). Sites surveyed off Vangunu were along a submerged barrier reef with a crest at ~12 m. Sites surveyed off Gatokae were moderately protected from wave exposure by a headland, while additional sites were sampled on the protected leeward side of smaller, adjacent islands (Kicha, Malimali, Mbulo). The reef surveyed at Kicha has been informally protected from fishing since 2010, although only two deployments were completed here so protection status was not factored into the analysis.

While the particulars of social context vary, all of the sites are held under CMT by associated tribal groups, and usage rights are broadly dispersed across tribe members. Usage rights for subsistence and artisanal fisheries are broadly open access, while large-scale commercial operations (e.g., bait fishing operations during the late 1990s; c.f., [14]) are negotiated at the tribal level. Several communities adjacent to the survey sites belong to the Seventh Day Adventist church, meaning there are edicts against the harvesting and consumption of shark, while others have customary links to sharks and do not harvest for those reasons. Because of the physical location of the communities (adjacent to the reefs), it is likely that those on Kolombangara are better able to monitor and restrict access to reefs when compared to Parara and Gizo.

### Sampling techniques and video analysis

#### Stereo-BRUV surveys

We used stereo-BRUVs to quantify reef shark abundance, biomass and habitat at the four locations across the Western Province of the Solomon Islands (Fig 1). Baited video is an effective tool for sampling the relative abundance of reef sharks [19,25,26]. At each location, sampling sites were randomly stratified along the barrier reef edge, with five stereo-BRUVs haphazardly deployed on coral-reef habitat at random depths between 10 and 25 m. The location of each BRUV replicate was not randomly assigned due to a lack of prior knowledge (i.e. bathymetry) for the locations surveyed. Adjacent deployments were separated by a minimum of 500 m to reduce the likelihood of sharks moving between replicates. Systems were left to film for 60 minutes, with bait consisting of 1 kg of skipjack tuna (*Katsuwonus pelamis*) in a plastic-coated wire mesh basket that was suspended 1.2 m in front of the two cameras. The tuna was chopped into small pieces to promote dispersal of the bait plume. For details and design of stereo-BRUV systems, see Harvey and Shortis [34]. A total of 108 hr of stereo-BRUV footage was collected across the Western Province, with 28 hr in Parara/Gizo, 24 hr in Kolombangara, 30 hr in Vangunu and 36 hr in Gatokae (Fig 1).

#### Stereo-DOV surveys

Stereo diver operated video (stereo-DOV) is more effective for sampling herbivorous and invertivore species (which make up a large proportion of reef shark diets [35]) when compared to BRUVs, so were used to survey reef fish and provide an estimate of prey density across the same locations (Fig 1). All sampling sites were randomly stratified along the barrier reef edge, targeting coral-reef habitat. At each site six 5 m by 50 m belt transects separated by 10 m were completed at depths between 8 and 12 m (to approximately match the average depth of BRUV surveys), following the methods outlined in Goetze et al. [31]. Transects took an average of 2 minutes and 3 seconds (± 1 sec SE) to complete.

The stereo-BRUVs and DOV systems in this study used GoPro Hero3+ Silver edition cameras mounted 0.7 m apart on a base bar inwardly converged at seven degrees. This allowed for a standardized field of view from 0.5 to 10 m for both systems [36].

#### Calibration

Stereo-video imagery was calibrated using the program CAL (http://www.seagis.com.au/bundle.html), following the procedures outlined by Boutros et al. [37]. This enabled measurements of the distance to, and angle of, the fish from the centre of the camera and standardisation of the area surveyed. Individuals further than 10 m in front of (determined by the minimum visibility) or 2.5 m to the left or right of the stereo systems were not recorded.

#### Video analysis

Identification and relative abundance estimates of sharks, rays and reef fish species were made by viewing video footage in the program EventMeasure (http://www.seagis.com.au/event.html). A MaxN was recorded for each shark and ray species observed during each 60 min BRUV deployment and was used as a measure of relative abundance. For the DOVs, all fish species observed within the 5 x 50 m transect were counted, identified to the lowest taxonomic level possible and measured. The fork length of fish and sharks was measured using stereo footage in EventMeasure and used to calculate biomass (kilograms) with length-to-weight regressions from FishBase [38]. Procedures for video analysis followed Goetze et al. [31], and data were extracted from EventMeasure software and checked following Langlois et al [39].

Broad-scale habitat and vertical relief were analysed in the program TransectMeasure (http://www.seagis.com.au/transect.html) following the method outlined in McLean et al. [40]. A 5 x 4 grid was overlaid on a high definition image for every individual BRUV deployment. The dominant habitat type and relief was characterised within each rectangle using the CATAMI classification scheme [41]. Habitat was selected from the broad habitat types: hard corals, macroalgae, unconsolidated (sand/rubble), consolidated (rocky bottom) and soft corals. Grid rectangles that were oriented to open water were classified as ‘no biota’ and removed before analyses. If grid rectangles were not marked as open water, an estimate of relief was also made and categorised from 0–5 based on the scheme in Wilson et al. [42], providing the mean relief for each deployment. Data were extracted from TransectMeasure software and checked using R code available in Langlois [43].

### Species selection

The three species of reef sharks observed, *Carcharhinus melanopterus*, *Triaenodon obesus* and *Carcharhinus amblyrhynchos*, were combined for statistical modelling as sample sizes were too small to undertake single species analysis. Rays were also removed from statistical modelling due to low numbers of individuals. To calculate prey biomass, fish families that were known to be the prey of reef sharks were extracted from the stereo-DOV data and included: Acanthuridae, Balistidae, Carangidae, Chaetodontidae, Haemulidae, Holocentridae, Labridae, Lethrinidae, Monacanthidae, Mullidae, Pomacentridae, Scaridae, Scombridae and Zanclidae [44–47]. Fishes of all sizes were included in calculations of prey biomass, as 99.5% were below 50 cm in length and therefore likely to be predated upon.

### Data analysis

The influence of location, habitat, prey density and depth on the relative abundance and biomass of reef sharks (as derived from MaxN) was investigated using generalised additive mixed models (GAMMs; [48]). To accommodate for over-dispersion and correlation in the data, we extended the application of this class of models by including sites as a random effect [49]. Model selection was based on Akaike Information Criterion (AIC; [50]) and AIC weights (wAIC; [51]). A full subsets method was used to fit models of all possible combinations up to a maximum of three variables to prevent overfitting. Models containing variables with correlations >0.28 were excluded from the analysis to eliminate strong collinearity in line with recommendations from Graham [52]; this can cause problems with over-fitting and make interpretation of statistical results difficult. Models with AIC values that differ by less than two units show weak evidence for favouring one over the other [53,54]. The best model was therefore the one with the fewest variables (most parsimonious) and within two AIC units of the lowest AIC value [54]. The wAIC, which represents probabilities or weights of evidence for each model, was used to facilitate interpretation of the best models. Relative support for each predictor variable was obtained by calculating the summed wAIC across all subsets of models containing that variable to obtain its relative importance [54].

Prior to analyses, two habitat categories (macroalgae and soft corals) were removed due to their limited coverage, and sand/rubble was excluded due to strong collinearity with the category reef. Models were fitted to untransformed abundance data using a Tweedie error distribution [55]. A Tweedie model is an extension of compound Poisson model derived from the stochastic process where a gamma distribution is used for the counted or measured objects (i.e., number or mass of fish) and has an advantage over delta-type two-step models by handling the zero data in a unified way. All analyses were performed using the R language for statistical computing [56] with the package GAMM4 version 0.2e3 [57]. Because P values derived from GAMMs are usually approximates and typically too low [58], we used a weight of evidence approach to test for significance in the factors identified by the most parsimonious models. Univariate permutational analysis of variance and co-variance (PERMANOVA and PERMANCOVA; with 9999 permutations and dissimilarity matrices constructed with Euclidean distance) was used for significance testing and to explore pairwise comparisons between locations in the PERMANOVA+ add-on to PRIMER [59].

### Social surveys

As part of a wider project [60], fisher survey data were collected from fishers at four villages adjacent to the sites on Parara, Kolombangara, Vangunu and Gatokae islands. These villages only represent a subset of the population of fishers that may target sharks in our survey areas and there are additional villages, as well as non-indigenous fishers, who are known to target sharks, which were not captured by this sample.

There were two primary survey methodologies. Catch per unit effort (CPUE) surveys were conducted with fishers at the four sites between February and October 2016. Following the format presented in Albert et al. [61], surveys were conducted approximately weekly by trained community researchers. The community researchers weighed and measured all fish and invertebrates brought back to the village, and noted details of the catch (time spent fishing, location, method). We also conducted semi-structured interviews with five expert fishers at each site in March 2017, to provide context for the shark survey data. These informants were asked three broad questions that surveyed their perceptions of shark fishing on reefs managed by the community (S1 File).

### Ethics statement

All human subject research was conducted under consent from community leaders and was approved by the American Museum of Natural History’s ethics board. Animal ethics for fish surveys were approved by the University of Western Australia, Animal Ethics Committee under approval number RA/3/100/1161.

## Results

A total of 163 sharks and rays were observed on the stereo-BRUVs, across 8 different species: *Carcharhinus melanopterus* (n = 71), *Triaenodon obesus* (n = 39), *Carcharhinus amblyrhynchos* (n = 31), *Aetobatus ocellatus* (n = 18) *Negaprion acutidens* (n = 2), *Taeniura lessoni* (n = 1), and *Urogymnus granulatus* (n = 1). On average, 1.33 (SE = 0.12) reef sharks were recorded per 60 minute replicate. The largest shark observed was *N*. *acutidens* at 209 cm and the smallest was *C*. *amblyrhynchos* at 56.7 cm (fork lengths). The average fork length across all locations for *C*. *amblyrhynchos* was 77.2 cm (SE = 27.8), *C*. *melanopterus* 80.9 cm (SE = 12.2) and *T*. *obesus* 88.6 cm (SE = 16.2).

**Fig 2:**
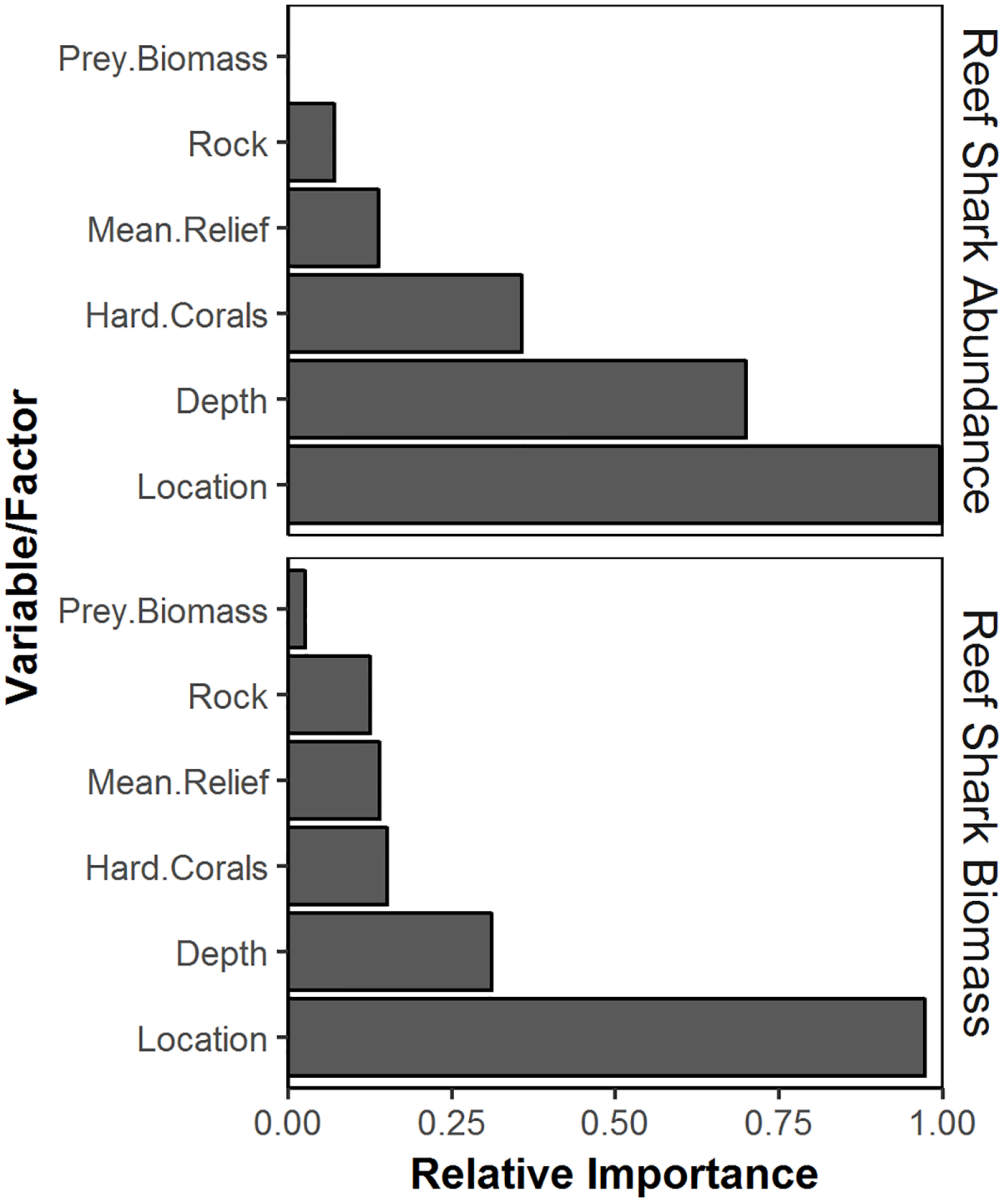
Importance scores for each explanatory variable in predicting the abundance and biomass of reef sharks.

**Fig 3:**
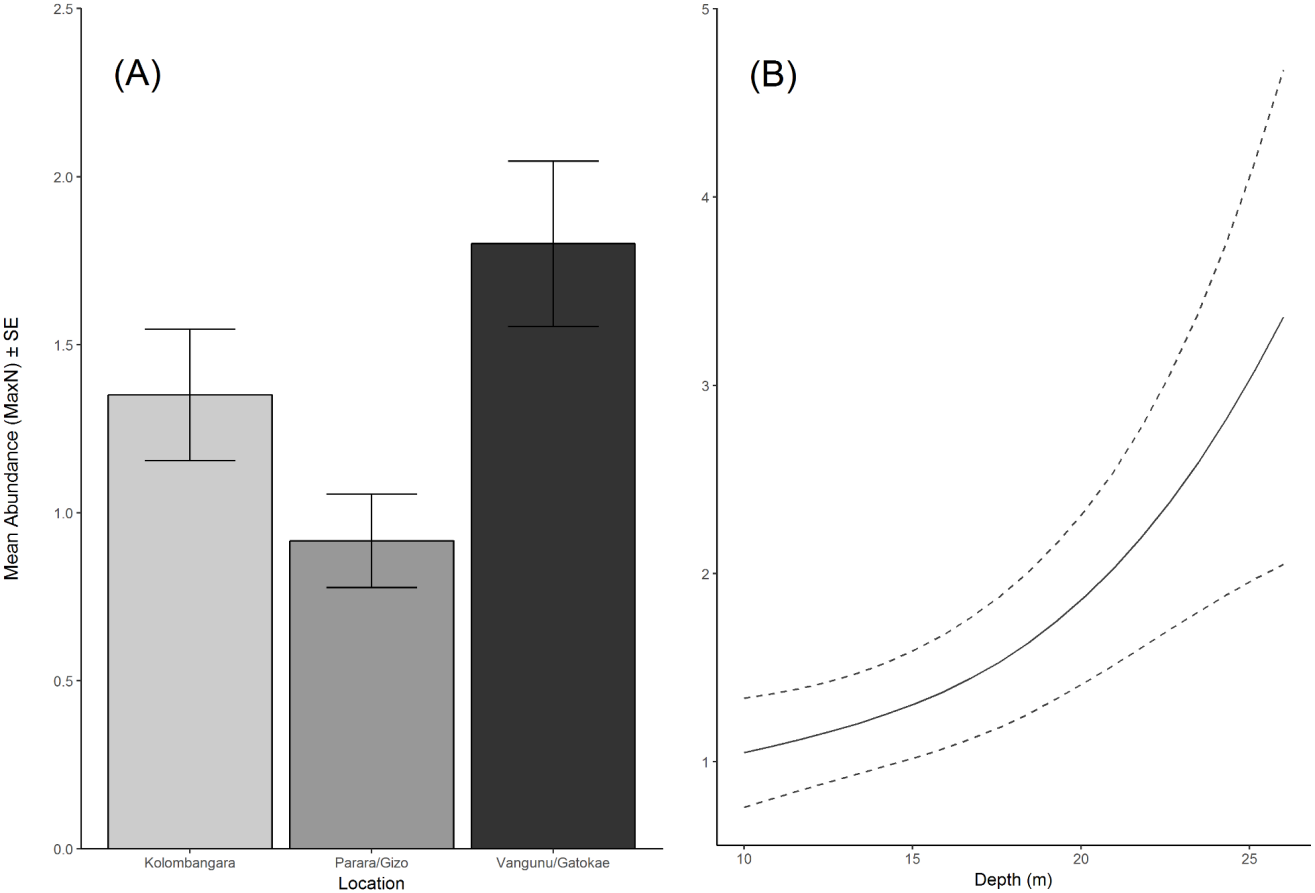
(a) The mean abundance of reef sharks per 60 minute replicate (MaxN) across locations and (b) The abundance of reef sharks (MaxN) across depth. Solid lines are fitted gam curves, with dashed lines indicating standard error confidence bands.

The best-fitted models utilising depth, habitat, location and prey biomass variables were generated for the abundance and biomass of reef sharks across the Western Province, of the Solomon Islands (Table 1). The variable importance plot illustrates the strength of these variables relative to each other (Fig 2). The most parsimonious model for the abundance of reef sharks included location and depth, which explained 22% of the variance in their distribution (Table 1). Location also occurred in the second top model, importance scores indicated that it was an important variable across all possible models (Fig 2, Table 1) and a significant difference was observed with PERMANCOVA tests (*P* < 0.001, Pseudo-F = 8.79, df = 2; Fig 3a). Depth was included in the top model and a significant increase in the abundance of reef sharks was observed with depth (*P* = 0.022, Pseudo-F = 2.93, df = 1; Fig 3b). Location was the only factor included in the top model and was the most important factor influencing the biomass of reef sharks (Fig 2, Table 1) with a significant difference observed with PERMANOVA tests (*P* = 0.046, Pseudo-F = 3.23, df = 2). The habitat variables (hard coral and rock), mean relief and prey biomass were not included in the top models for abundance or biomass (Table 1, Fig 2).

**Table 1:** Top GAMMs for predicting the abundance and biomass of reef sharks across the western province of the Solomon Islands from full subset analyses. Difference between lowest reported corrected Akaike Information Criterion (ΔAICc), AIC weights (ωAICc), variance explained (R2) and effective degrees of freedom (EDF) are reported for model comparison. Model selection was based on the most parsimonious model (fewest variables) within two units of the lowest ΔAICc as shown in bold.

Variable	Best models	ΔAICc	ωAICc	R2	EDF
Abundance	Location + Hard Coral x Location	0	0.308	0.163	7
	**Location +Depth**	**0.978**	**0.189**	**0.216**	**6.63**
	Location + Mean Relief + Depth	1.17	0.172	0.213	7.75
Biomass	**Location**	**0**	**0.137**	**0.098**	**4**
	Location + Depth	0.185	0.125	0.078	5
	Location + Hard Coral x Location	0.232	0.122	0.103	7
	Location + Consolidated	1.111	0.079	0.101	5.6
	Location + Depth + Hard Coral x Location	1.495	0.065	0.119	8.18
	Prey Biomass	1.518	0.064	0.083	5
	Location + Hard Coral	1.669	0.06	0.092	5.52

**Fig 4:**
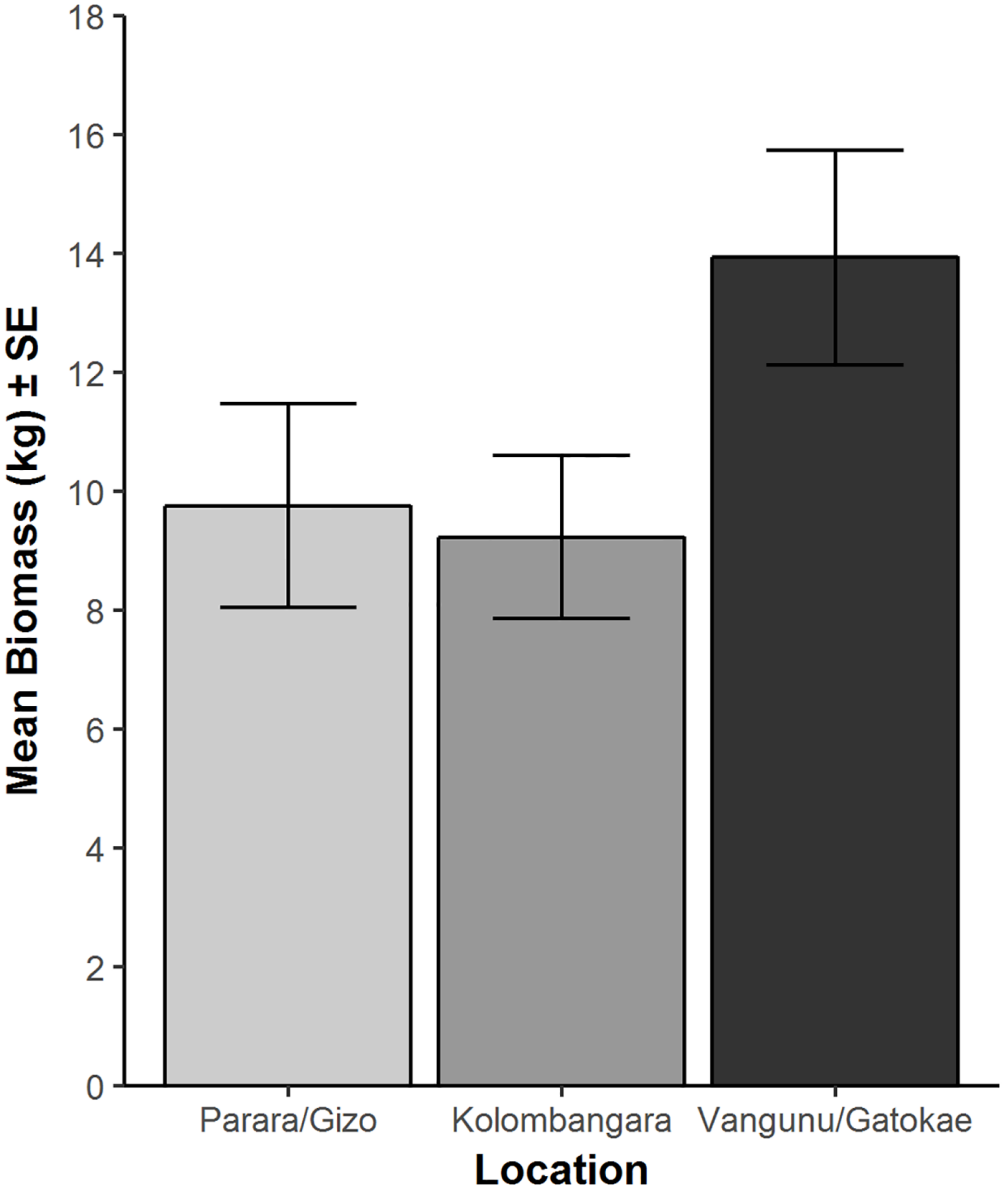
The mean biomass of reef sharks and rays per 60 minute replicate (MaxN) across locations.

Pairwise comparisons between locations showed that the abundance of reef sharks was significantly greater (~2 times) in the more remote locations Gatokae and Vangunu when compared to Parara/Gizo (*P =* 0.001, t = 4.23; Fig 3a). There was a 47% greater abundance of reef sharks observed at Kolombangara when compared to Parara/Gizo, although this was not significant (*P* = 0.079, t = 1.82). Pairwise comparisons revealed the average biomass at the more remote locations Gatokae and Vangunu was 43% greater than Parara/Gizo, although again was not significant (*P* = 0.089, t = 1.72; Fig 4).

### Survey data

CPUE surveys recorded 833 fishing instances across the four communities. Only one shark, a blacktip reef shark (*C*. *melanopterus*) on Parara Island, was recorded in the CPUE effort across all survey locations. This shark was kept for local consumption.

### Interview data

Semi-structured interview data provided additional context for the ecological surveys. Some fishers at Kolombangara and Parara said that they had seen shark fishers setting buoys in the past, and when buyers were present in Gizo they could be seen over 20 times a year. Fishers at both Vavanga and Parara noted that there had been little shark fishing activity over 2016 and 2017 because there had been no buyers of fins, though they did note there had been some fishing towards the end of 2015. On Gatokae and Vangunu, interviewees had seen shark fishermen occasionally in the past but did not think it to be a current threat. No interviewees perceived it to have been an issue over the past 3–5 years. No interviewees mentioned consumption of sharks for food, which is not surprising given that two of the communities belong to the Seventh Day Adventist church, whose teachings forbid the consumption of shark.

## Discussion

We provide the first assessment of the relative abundance, biomass and diversity of reef sharks and rays for the Solomon Islands where this information is historically lacking [32]. When compared to studies using the same methodology, our observation of 1.33 reef sharks per hour was above the average of comparable habitats in the western Indo-Pacific both inside and outside of no-take marine reserves in Fiji (Protected: 0.8, Fished: 0.3; [26]) and Indonesia (Protected: 0.8/0.6, Fished: <0.1; [30]), and well above other areas globally where severe overfishing has occurred (i.e. the Eastern Red Sea; <0.01 sharks per hour [29]). However, relative abundance estimates were lower than remote areas of the Pacific, where reef sharks have not been exploited (e.g. remote areas of New Caledonia >2.5 sharks per hour [33] and Palmyra Atoll; *C*. *melanopterus* >2 sharks per hour [62]). Combined, these results suggest that reef shark populations in the Solomon Islands are relatively healthy when compared to nearby countries, yet likely to have been impacted by fishing given they are not comparable to remote areas without fishing pressure.

The diversity of reef sharks (as observed using baited video) was dominated by three species: *C*. *melanopterus*, *T*. *obesus* and *C*. *amblyrhynchos*. These coral-reef Carcharhinidae are found commonly throughout the tropical Indo-Pacific [63], and while ongoing collapses of reef shark populations have been documented in some areas [64], this varies greatly depending on the location [8,27]. We found that location was the most important factor influencing reef sharks across the Solomon Islands, which was primarily driven by a significantly greater abundance and biomass of sharks in the more remote locations of Gatokae and Vangunu.

The biomass of reef sharks at Kolombangara was similar to Parara/Gizo, and combined with abundance results, suggests that sharks were more numerous but not larger. It is common to record different results between abundance and biomass with finfish data [65–67]. Here, the increased abundance of sharks but not size within the customary managed area of Kolombangara may be due to the fact that larger sharks generally have larger home ranges [68–70]. This would increase the time spent in areas where sharks are more easily targeted, making these larger sharks more vulnerable to fishing mortality when compared to smaller sharks with a higher site-residency [71].

Previous studies have shown that prey biomass [26,72] and coral-reef health [27,73,74] are important drivers for the abundance of reef sharks. These factors were not the primary drivers for reef shark abundance or biomass observed in the Western Province of the Solomon Islands, and instead location was the most important factor. It is likely that the difference in locations was primarily driven by proximity to increased fishing pressure around the Gizo area, however further work is needed to confirm this. Studies in the Caribbean have shown that fishing pressure can be a more important driver of reef shark abundance when compared to environmental factors [25]. While we would expect both prey density and habitat to have an impact on shark populations in the Solomon Islands, it is likely that fishing pressure is having a greater effect and a relatively healthy coral-reef environment throughout the Western Province has contributed to this result. We also acknowledge that we have only used finfish density as a proxy for reef shark prey, and while benthic fishes make up the largest proportion of their diet [46,47,75], a recent review suggests that reef sharks also consume a large proportion of cephalopods and crustaceans [35,72].

There are a number of physical and structural differences that vary across regions [76] that may have explained additional variation in reef shark abundance and biomass. For example, studies have shown that temperature [77], productivity [8], wave energy and broad-scale structural complexity [78] can all impact on the distribution of reef sharks and fish. These factors were not examined here as detailed information was not available (i.e. fine scale bathymetry) or they were expected to vary across the different species of sharks that were grouped together (e.g. broad-scale habitat preferences for *T*. *obesus* vs. *C*. *amblyrhynchos*).

Shark fishing for fins has a long history in Western Province [13], and at certain times is likely to have occurred around all sites covered by this survey. Small-scale, inshore shark fishing operations are permitted under Solomon Island laws, while commercial offshore operations are banned. However, shark fin buyers do occasionally operate in Gizo and the capital Honiara. The sites off Kolombangara, Parara and Gizo islands are close to potential buyers in Gizo (between 5 and 25 km) and fishers on Parara and Gatokae islands have reported sporadic, small-scale longline operations targeting mainly grey reef sharks close to the reef edge.

The lack of sharks in the CPUE and interview data across all survey locations suggests local fishers preferentially target shallow-water estuarine and reef fish, and keep only the occasional shark for local consumption or sale [12]. It is possible that this is a driver of the relatively healthy population of reef sharks observed in these surveys. The low level of exploitation for reef sharks across our sites may also be due to the cultural importance of sharks in parts of the Solomon Islands, where they are sometimes regarded as embodiments of gods, guardians and protectors [32,79,80]. However, more recently the traditional symbolism and values that were associated with sharks and rays has begun to lose its significance and international markets for shark meat and fins are believed to occur in small-scale fisheries throughout the Solomon Islands [32]. In terms of the potentially greater abundance of sharks in Kolombangara, it is notable that the Gizo and Parara sites are close to large populations of non-indigenous fishers who are known to target sharks. This is further supported by the fact that the reefs in Kolombangara are directly adjacent to communities and are therefore more actively and easily policed. It is possible that fishing pressure from non-local fishers is impacting on shark abundance at some of these sites, though further targeted research is needed to assess this.

We provide the first assessment of reef sharks and rays across the Western Province of the Solomon Islands using stereo-BRUVs. While reef shark abundance and biomass were relatively high when compared to neighbouring countries in the Indo-Pacific (although below those of remote areas), a significantly lower abundance of reef sharks at sites around Parara and Gizo indicate that fishing pressure may be impacting on populations in these areas. A broader scale study across a larger range of locations and habitats (i.e. lagoons and mangroves) would provide important information to assist in the conservation and management of sharks and rays across the Solomon Islands. Sampling effort in this study was limited to a snapshot of the relative abundance and biomass of sharks and rays, however, these levels are unlikely to vary significantly across seasons due to the high site fidelity of reef sharks [77,81,82]. Our results provide a baseline that suggests reef shark population in the Solomon Islands are still relatively unexploited, providing an opportunity for improved conservation and fisheries management to protect existent populations.

## Supporting information

S1 File Questions asked during semi-structured interviews. (DOCX)

S2 File Raw data from BRUV and DOV surveys. (ZIP)
