# Supplementary material for: Drivers of reef shark abundance and biomass in the Solomon Islands
Source: PLoS One. 2018 Jul 30;13(7):e0200960. doi: 10.1371/journal.pone.0200960 (PMC6066198; doi:10.1371/journal.pone.0200960)
Supplement: S1 File — (DOCX) [file pone.0200960.s001.docx]

Semi-structured interviews were conducted using the following three questions that led to discussions around who was doing the fishing, what was driving the fishing pressure, where people were fishing and what for:

- Have you seen people fishing for sharks on [*insert name of reefs close to village?*]
  - If yes, how often, when do you see them?
- How many times a year do you see people fishing for sharks on *[insert name of reef close to village*]?
  - If yes, what techniques do they use?
- Have you seen people from here [i*nsert village name*] fishing for sharks?

Noting all interviews were preceded by an informed consent procedure which:

- Gave information about the project and what data was being used for, including why we wanted to speak with the interviewee (i.e., because they were a fishing expert)
- Informed participants that it would be confidential - i.e., their names would not be attached to any reported data
- Gave information on how data would be fed back to the community through the wider biocultural program
- Allowed participants the option of opting out at any stage
- Asked consent to continue.
